# Adherence to oral anticancer chemotherapy: What influences patients’ over or non-adherence? Analysis of the OCTO study through quantitative–qualitative methods

**DOI:** 10.1186/s13104-015-1231-8

**Published:** 2015-07-04

**Authors:** Aurélie Bourmaud, Emilie Henin, Fabien Tinquaut, Véronique Regnier, Chloé Hamant, Olivier Colomban, Benoit You, Florence Ranchon, Jérôme Guitton, Pascal Girard, Gilles Freyer, Michel Tod, Catherine Rioufol, Véronique Trillet-Lenoir, Franck Chauvin

**Affiliations:** EMR3738, Therapeutic Targeting in Oncology, Claude Bernard University, Lyon, France; Public Health Department, Hygée Centre, Lucien Neuwirth Cancer Institut, Inserm, CIC1408, 108 bis avenue A. Raimond, 42 270 Saint Priest en Jarez, France; Medical Oncology Department, Teaching Hospital, Lyon-Sud University, Lyon, France; Oncologic Pharmaceutical Department, Lyon-Sud University Teaching Hospital, Lyon, France; Pharmacology-Toxicology Laboratory, Hospices Civils de Lyon, South Biology Center, Lyon, France; Jean Monnet University, Saint-Etienne, France

**Keywords:** Oral chemotherapy, Capecitabine, Adverse events, Adherence, Profiles, Quantitative–qualitative mixed method

## Abstract

**Background:**

Numerous oral anticancer chemotherapies are available. Non-adherence or over-adherence to these chemotherapies can lead to lowered efficacy and increased risk of adverse events. The objective of this study was to identify patients’ adherence profiles using a qualitative–quantitative method.

**Methods:**

A capecitabine treatment was initiated for 38 patients with advanced breast or colorectal cancer. At inclusion, information on patients’ beliefs was reported using a questionnaire. Later, Information on patients’ relation to treatment was obtained from a sub-group during an interview with a sociologist. Questionnaires were analyzed using Multiple Classification Analysis to cluster patients. Treatment adherence was evaluated by an electronic medication event monitoring systems (MEMS caps) and then correlated with patient clusters. Interviews were analyzed to complete and explain results.

**Results:**

38 patients were enrolled between 2008 and 2011 and completed the questionnaire. Twenty had adherence measured with MEMS caps all along treatment. Between 4 and 6 months after inclusion, 16 patients were interviewed. Patient profile B (retired, with a regular life, surrounded by a relative’s attention to drug adherence, with a low educational level) was statistically associated with adequate adherence (p = 0.049). A tendency for lower adherence was observed among more highly educated patients with an irregular, active life (NS). All patients taking capecitabine demonstrated a risk of over-adherence, potentiating side effects.

**Conclusions:**

These encouraging primary results suggest that further studies should be undertaken and that educational programs tailored to patient profiles should be evaluated to enhance adherence for those who need it and to empower all patients to manage treatment side effects.

**Electronic supplementary material:**

The online version of this article (doi:10.1186/s13104-015-1231-8) contains supplementary material, which is available to authorized users.

## Background

Oral anticancer therapy is being increasingly used every year, in particular cytotoxic agents, and more recent targeted therapies, are often administered orally. The main advantages of oral administration are the fewer hospitalisations and the autonomy gained by patients. However, these advantages can be compromised since the patient has to be responsible for the ambulatory administration and monitoring of these drugs. This raises two areas of potential concern: the patient’s adherence and the occurrence of unknown side effects. Capecitabine is a fluorouracil (5 FU) prodrug and is prescribed orally for the treatment of advanced and metastatic breast, colorectal and gastric cancers. Several studies have reported patient adherence rates for capecitabine that vary from 58 to 100%, mainly dependant on the measurement method [1–11]. In most studies, non-adherence rate remains at about 20–25% [2, 3, 5, 7, 11]. The reasons for non-adherence can be grouped under three main themes [12]: Personal factors (belief in the treatment, emotional state); treatment factors (complexity of treatment, side effects, costs) and healthcare provider-related factors (relationship with healthcare professionals, prescribing practices). Few factors have been identified to explain non-adherence with capecitabine: the number of co-medications, and the number of side effects [3, 9, 10, 13]. Adherence with capecitabine is relatively high compared with other oral cancer chemotherapies [14]. It has been suggested that there is only one specific group of patients who are non-adherent and that adherence-enhancing intervention should be targeted to this group only [7, 9, 13]. The difficulty is to be able to identify them early and propose the interventions to them.

The second area of concern for oral chemotherapy is the monitoring of side effects. Capecitabine is responsible for gastrointestinal and dermatologic side effects that can lead to life-threatening toxicity, which can be resolve by temporarily interrupting treatment or modifying the dose. However, serious side effects still occur with capecitabine [15] and these are often related to over-adherence since, when side effects occur, there is no medical surveillance or management of doses, the patients continue to take the drug [16, 17]. This over-adherence has been observed, particularly, with capecitabine [2, 3, 6, 8, 18]. There is, therefore a need to develop interventions that can enable quick recognition of these side effects and their timely, adequate management.

The objective of the OCTO study (Observance à une ChimioThérapie Orale—Adherence to oral chemotherapy) was to model adherence of ambulatory patients treated with capecitabine. An ancillary study (OCTOquali) within the main OCTO study was conducted assuming that there were profiles for patients to determine their non-adherence or over-adherence. The identification of these profiles would allow early tailored, patient education programmes for the adequate administration and monitoring of their care to be proposed to the patients.

The main objective of the OCTOquali study was to define patient profiles that will predict and prevent poor adherent behaviour.

## Methods

### Study design

The OCTOquali study was a prospective monocenter cohort study that recruited patients initiating an oral capecitabine treatment between November 2008 and September 2011. The patients answered a questionnaire at inclusion and a sub-group of patients was interviewed 4–6 months after the inclusion in the study to explore their beliefs and behavior related to their treatment. These results were analyzed to identify sociological profiles among the patients and compare these profiles with the patients’ adherence data.

### Participants

Patients were recruited in the medical oncology department at the Lyon-Sud university hospital of Lyon (France). Consecutive adults with colorectal cancer at a resected or metastatic stage or breast cancer at a locally advanced or metastatic stage, with an indication for an ambulatory capecitabine treatment, were screened by the oncologists and recruited for the study. Written informed consent was obtained from each participant. The study was approved by Lyon’s South-East ethical committee number 4 (approval number 2008-004097-41).

### Setting

Patients were planned to be followed during six treatment cycles. The treatment dose was based on their body mass index (BMI), and prescribed as a combination of 150 and 500 mg tablets. Treatment was taken as morning and evening doses, at exactly 12 h intervals, within 30 min after a meal. Tablets could not be split to help swallowing. A treatment cycle was 14 days of treatment followed by a ‘rest’ week. Half the patients (N = 20) were given a bottle of capecitabine with a MEMS cap (Medication Electronic Monitoring Systems, Aardex), to measure their adherence electronically. At inclusion, the patients completed an initial questionnaire exploring socio-economic characteristics and beliefs about their new oral chemotherapy, including self-predicted adherence. 4 to 6 month after inclusion, the patients were proposed a semi-structured interview, conducted by a sociologist to evaluate their representations and behavioral adaptations made since initiating capecitabine treatment.

### Measure of adherence

Adherence was measured during all treatment intake (at most 6 cycles) using the timing of each opening of the bottle with MEMS caps for 20 patients: This was used to define three types of adherence. Patients with an opening time variability of >1 h standard deviation were considered to have poor adherence, those with an opening time variability of <15 min were considered to have high adherence and patients with a variability between were considered to have adequately adherence. Those cut-offs were defined as clinical relevant by a multidisciplinary team (two oncologists and one sociologist). Missed doses were very rare in this cohort so we did not measured adherence this way.

### Questionnaire

The questionnaire was constructed by a sociologist, an oncologist and a methodologist. The questionnaire was tested on five patients and modified to improve its clarity. The questionnaire, which contained 32 closed questions answered directly by the patients, focussed on their perceptions and expectations (Additional file 1). It examined socio-economics conditions, relationship with their oncologist, beliefs in treatment efficacy and toxicity, expected adherence, expected changes in behaviour related to taking oral chemotherapy.

### Interview

Face-to-face interviews were proposed to patients after the end of the adherence measurement period (6 cycles at most). Patients were randomly recruited for the interviews, sampling continuing until data saturation was achieved. Interviews were conducted face-to-face with a sociologist, lasted at least 1 h and investigated those topics : (i) the declared adherence behavior and perception (ii) the patient understanding of his treatment and its management (iii) the place of relatives in treatment management (iv) patient’s adverse event declaration and management.

See the Additional file 1 for more information on “Methods”.

### Statistical analysis

Patients’ answers were first analysed descriptively by frequencies (percentages) and medians (interquartiles). Then they were analysed with a multiple classification analysis (MCA) followed by an ascending hierarchical classification (AHC), which is an agglomerative hierarchical clustering procedure, to statistically and graphically identify patient clusters.

Adherence categories identified with MEMS caps were projected onto a MCA cluster map to graphically identify adherence clusters. Patients without adherence measured with MEMS caps were considered as having missing data. Then categories of adherence were compared with the whole clusters tendency using univariate analyses. All analyses were performed using SAS (version 9.1) with a significance threshold of p < 0.05.

The interviews were analysed with socio-analytical methods: the different elements of the discourse during each interview were connected and then underwent content analysis.

Clusters identified with the MCA methods were linked with the results of the interview analyses, to evaluate the concordance of common topics, to explain the MCA findings and to describe profiles of interest.

## Results

**Figure 1:**
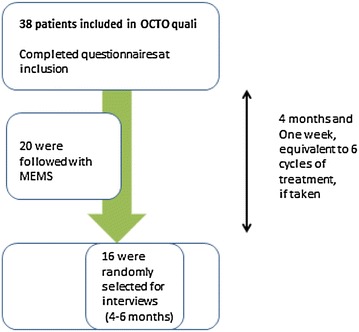
Flow chart of the patients included in the OCTOquali cohort.

38 patients were included in the OCTOquali study. 20 of them were followed by MEMS during all the treatment intake, until the first event occurred: either treatment discontinuation or the end of the 6 cycles. 16 patients were included after the 6 cycles period (with or without treatment), for the interviews, one by one, until data saturation was reached. 4 patients refused to conduct the interview (Figure 1). The main reason given for not completing the interview is not being available.

### Questionnaire analysis

The average age of the 38 patients who completed the questionnaire was 59 years (Table 1). The majority of responders included were women (n = 35; 92%), had breast cancer (n = 33; 87%), lived in town (n = 26; 68%). Only 32% (n = 12) still had at least one child living at home and 21% (n = 8) had a professional activity.

**Table 1:** Patients’ characteristics

Characteristics, N = 38	Patients, n (%) or mean (±sd)
Socio-demographic characteristics	
Age (years)	58.6 (11)
Gender	
Women	35 (92.1)
Men	3 (7.9)
BMI	
<25.5	10 (26.3)
25.5–28	18 (47.4)
>28	10 (26.3)
Smokers	6 (15.8)
Residence	
Town	26 (68.4)
Country	12 (31.6)
Living conditions	
Alone	11 (28.9)
As a couple without children	15 (39.5)
As a couple with children	12 (31.6)
Educational level	
Primary school	12 (31.6)
Secondary school	16 (42.1)
College	8 (21.1)
Occupation	
In activity	8 (21.1)
Retired	15 (39.5)
On sick leave	15 (39.5)
Actual or former profession	
Artisan or laborer	4 (10.5)
Intermediate occupation	23 (60.5)
Executive	8 (21.1)
Does not apply	3 (7.9)
Disease related characteristics	
Type of cancer	
Breast	33 (86.8)
Colon	5 (13.2)
Length of oral chemotherapy	
<3 cycles	13 (34.2)
≥3 cycles	25 (65.8)
Reason for discontinuation	
Toxicity	6 (15.8)
Disease progression	9 (23.7)

The patients’ feelings about their treatment, taking the treatment and their relationship with their oncologists are summarized in Table 2. Eight (21%) patients said they knew that an alternative treatment, involving intravenous administration in hospital, was available. Eleven (29%) patients knew perfectly well the dose they had to take. They said that there were fewer side effects with oral treatment and generally ambulatory treatment gave more advantages (autonomy, less anxiety, fewer side effects—all 71%) than disadvantages (the need for rigor of administration, blood tests and loneliness—all 51%). When they were asked about the way their oncologist provided information, the patients said they were satisfied; only 3 (8%) patients said that information related to side effects was insufficient. The patients said they wanted to manage their new treatment; 13 said they wanted to get more information about the treatment, 10 on the internet. The majority of patients, 26 (68%), said they wanted to adjust their living style to take into consideration the treatment administration. Eleven (29%) patients said they could miss a dose and 4 that they could miss it voluntarily. Six patients said that it was not a problem to miss a dose. Sixteen patients said the thought that missing a dose was dangerous and 16 said they did not want to answer. If a serious side effect occurred, 33 patients (87%) said they would continue the treatment, with or without consulting their oncologist.

**Table 2:** Responses to the questionnaire

	Responders, N = 38
Cancer treatment	
Already had taken oral chemotherapy	13 (34)
Knew about the choice between oral/intravenous	8 (21)
Knew the prescribed dose	
Perfectly well	11 (29)
Relatively well	22 (58)
Not well	4 (11)
Had another treatment for their cancer	10 (26)
Takes a treatment for side effects related to previous cancer treatment	8 (21)
Takes a treatment for another condition	21 (55)
Feelings related to capecitabine	
Advantages of the oral route, according to the patient	
Autonomy	22 (58)
Less anxiety	1 (3)
Fewer side effects	4 (11)
No advantages	3 (8)
Disadvantages of the oral route, according to the patient	
The lack of rigour in drug administration	16 (42)
Blood test	2 (5)
Loneliness	2 (5)
No disadvantages	4 (11)
Relationship with the oncologist	
Explanations given by the oncologist about:	
The treatment (organisation, administration) were insufficient	1 (3)
The treatment side effects occurrence were insufficient	2 (5)
Management of the treatment side effects were insufficient	3 (8)
Questions asked to the oncologist about the first prescription capecitabine	
Side effects	16 (42)
Loss of hair	2 (5)
Efficacy	2 (5)
Treatment duration	2 (5)
Intention to obtain more information elsewhere	13 (34)
Other clinician	3 (8)
Internet	10 (26)
Expected changes in daily life due to capecitabine	
Will have to be organised around the treatment administration	26 (69)
If side effects appear:	
I will stop the treatment	5 (13)
I will consult a clinician	23 (6A)
I will continue the treatment whatever	4 (10.5%)
Opinions about adherence	
I think that it is alright to miss a dose	11 (28.9)
I think that it is alright to stop voluntarily	4 (10.5%)
I think that missing a dose is dangerous	
Yes	16 (42.1%)
Did not want to answer the question	16 (42.1%)
No	6 (15.8%)

The numbers are n (%).

### Clusters identified by the MCA and AHC using questionnaires’ responses

Eighteen categorical variables (binary or ordinal) were selected for their pertinence to characterize patients: socio-demographic variables; relationship to the treatment; relationship with their oncologist and perception of treatment adherence. Using these variables the MCA generated a 2-dimension map representing the independent variables (Additional file 1: Figure S1 A). This graphical representation was used in AHC to define patient clusters (Additional file 1: Figure S1 B). The closest observations were merged to form one cluster, and the process reiterated until three clusters appeared (Table 3). Patients in cluster A were had a higher educational level and were active professionally, with an executive occupation, they lived in couples with children and intended to get more information about their treatment. Those patients generally thought that they could miss doses. Cluster B was composed mostly of retired people, with a lower educational level who thought that being non-adherent was dangerous. Cluster C was composed of urban patients, slightly younger, with a higher educational level than patients in cluster B, were on sick leave and had already taken oral chemotherapy at home, and they said it was not possible to miss a dose.

**Table 3:** description of the three clusters identified by CMA and AHC

Clusters, N = 38	P value
Cluster A, N = 16	
Educational level: college	0.0003
Actual of former profession: executive	0.048
Reason for discontinuation: disease progression	0.002
Length of oral chemotherapy ≤3 cycles	0.003
Intention to get more information elsewhere	0.02
Thought a dose could be missed during treatment	0.02
In couple with children	0.049
Cluster B, N = 9	
Occupation: retired	<0.0001
Educational level: primary school	0.0001
Thought that missing a dose was serious	0.02
BMI <28	0.042
Cluster C, N = 13	
Educational level: secondary school	0.003
No discontinuation of protocol	0.004
Length of oral chemotherapy ≥3 cycles	0.014
Did not know the prescribed dose well	0.009
Occupation: sick leave	0.01
Residence: town	0.03
Did not think that a dose could be missed during treatment	0.03
Already had had oral chemotherapy	0.04
Did not know there was a choice between oral/IV treatment	0.04

### Adherence results with MEMS records

Among the 20 patients whose adherence was measured electronically, adherence was globally estimated as concordant with the prescription since only 23 missed doses were recorded for 2,272 predicted doses (99% adherence). Two patients were classified as being less adherent (10%), 4 as being highly adherent (20%) and 14 (70%) as being adequately adherent, based on the mean inter-dose interval, which was estimated to be 12.15 h (Additional file 1: Figure S2). The standard deviation for the morning dose was estimated to be 1.003 h and 1.06 for the evening doses.

### Cluster profile and adherence behaviour association

**Figure 2:**
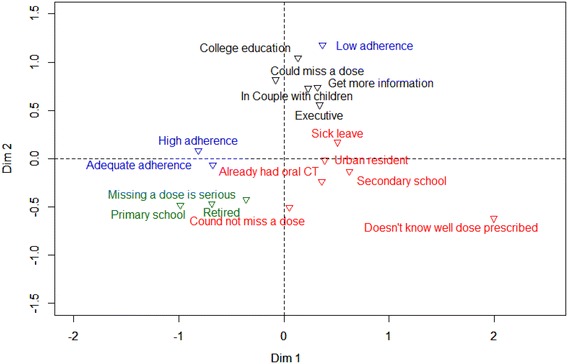
Graphical representation of the three clusters identified by the ascending hierarchical classification (AHC), with projection of adherence data, obtained for the 20 patients controlled with MEMs caps.

When the three adherence categories were projected onto the MCACA map (Figure 2), the patients classified as being low adherent were graphically located close to cluster A. The other two categories of adherence (highly adherent and adequate adherent) were located near cluster B. When concordance between clusters and adherence location was tested, cluster B was significantly associated with adequate adherence (p = 0.049, Figure 2). The associated for the other two were not statistically significant.

### Comparison of interview results and MCA results: confirmation of profiles by a mixed method

Detailed results from the interviews are reported in the Additional file 1.

Interviews identified two different profiles based on feelings and behaviour related to risk of non-adherence to capecitabine (Additional file 1: Table S1). MCA showed that one profile was similar to cluster A and the other was similar to both clusters B and C. Patients who had active lives, with an active partner and children still at home, said that was difficult and constraining to adjust to the capecitabine administration. They said their relatives were too busy and not available to share their adherence issue, leaving the patient alone with this responsibility. This loneliness generated anxiety among those patients, who were worried about forget to take a dose. These patients liked the idea of supervised IV administration in a hospital setting, in order to decrease their loneliness by being about to talk with healthcare providers. In contrast, patients with a regular life, and supportive and available relatives said that capecitabine administration was easy to organize and they tolerated it well. They shared the responsibility of adherence with their partner who helped them with the timing of the doses. They said they were satisfied and were not anxious about taking oral chemotherapy at home.

We were unable to identify profiles of patients for their feelings and behaviour related to side effects and over-adherence. They all had a great expectation in the efficacy of this treatment. This hope surpassed any criticism towards capecitabine intake and any side effects occurrence. What patients declared to fear at most was dose reduction. So face to side effects occurrence, all patients developed their own management strategy, in order to continue to take the treatment whatever the cost.

## Discussion

This study combined complex descriptive statistical methods and a sociological approach to identify, explain and confirm two adherence profiles: adequately adherent (Cluster B) and less adherent (Cluster A). Patients with no professional or family obligations, retired, and leading a calm and scheduled life, were found to be adequately adherent. Adherence was enhanced when the patient had a relative who helped them manage their treatment with them. These patients had a lower educational level, and followed the prescription strictly, for fear they would forget a dose. In contrast, patients with an irregular, active life, with family and professional obligations were more likely to be non-adherent. To keep a schedule when unexpected and external events can occur is more difficult. These patients experienced loneliness and thought they were benefit from having contact with a health professional, to share the burden of treatment, even that meant replacing the oral treatment with intravenous treatment. These patients had a higher educational level and were more aware that they could miss a dose of their treatment. This highlights that they are more in need of special support to help them manage their treatment.

The results showed that patients under oral capecitabine generally adhere to their treatment, with those in Cluster A being occasionally non-adherent (Cluster A). The occurrence side effects and their management was the main issue for all the patients, as they all reported having experienced side effects, often quite severe (62.5%). The interviews with the sociologist brought out the fact that some of these side effects were probably due to over-adherence: All the patients had high expectations about the efficacy of the capecitabine treatment, and were all ready to suffer from any kind of side effect, as long as the treatment was not stopped. Thus, they developed their own to strategies to support the side effects and were reluctant to tell their oncologist about them out of fear that the treatment would be stopped. This behaviour could lead to the side effects becoming more severe suggesting that there is a need for tailored support to teach patients how to manage the side effects.

Our adherence results are coherent with previously published results, i.e. 100% if adherence is defined as the number of forgotten tablets [1, 9]. However, when adherence is measured with more specific tools, some patients are less adherent than the others (from 40 to 9%, according to the studies [1, 2, 9, 10]), so our estimation of 10% of non-adherent patients is at the low end of this range, but remains consistent.

Some studies that measured over-adherence reported this to be between 4 and 44% [2, 6, 8]. In this study we did not specifically measure over-adherence, but rather investigated patients’ beliefs and behaviour related to over-adherence. All patients said they could be over-adherent during the interviews, giving a risk of over-adherence of 100%. This was supported by their misconception about efficacy and side effects reported on the questionnaire they completed when they received the prescription. Patients systematically overestimated the balance between the advantages and disadvantages of the oral treatment. They underestimated the risk of side effects. Even before starting the treatment, 10.5% of the patients said they would not stop treatment if side effects occurred. This misgiving increased over time, as was observed in the interviews.

Our results showed that there was a good relationship between the oncologist and patient; only three patients thought the explanations about side effects were insufficient and only one thought explanations about the treatment were insufficient). In addition, the results showed that the patients were motivated to be an active stakeholder in their care: 34.2% said they were going to find more information about the treatment, and 68.4% said they were going to organise their daily life around the treatment administration. These two positive attitudes should be used by healthcare providers to enhance effective management of treatment administration and monitoring by the patients. Patients appeared to be eager to become independent about their treatment, so a solution could be that their oncologist, that they trust could propose to them to participate in a programme to help them to manage their treatment. A therapeutic education programme would seem to be the most relevant intervention in this context. Therapeutic education programmes have been shown to be effective in improving treatment adherence in patients with non-oncological pathologies [19, 20]. It is recommended in the setting of oral capecitabine [2, 21] and would empower the patients.

The main limitation of this study is that only 38 patients were included, with only 20 of them having adherence measured with MEMS. This led to a lack of power, and to non-significant results for 2 of the 3 profiles identified. However, the AHC and MCA analyses enabled us to identify an adequate adherence profile. Also, graphically there seemed to be a close relationship between low adherence and Cluster A, even if this was not statistically significant. What’s more, the content analyses of the 16 sociologist’s interviews provided a wealth of information which was consistent with the quantitative results, and confirmed findings. Another limitation is the little number of patients with poor and high adherence. Thus it is difficult to say if the lack of association between those profiles and adherence behavior is due to lack of statistical power or if there is actually no association. The last limitation is that there was no objective measurement of over adherence and assessment of its relationship to the occurrence and severity of side effects, although it seems that this assumption is valid based on the patients’ interviews. The results from this study have now to be confirmed in a larger study. If confirmed, those profiles could be used to develop targeted educational interventions, with the objective of enhancing adherence and side effects management through empowerment of patients.

## Conclusion

In conclusion, this study, with a mixed quantitative–qualitative method, identified an adequate adherence profile for oral capecitabine. There also seems to be a specific low-adherence profile. The results show that over adherence and inadequate management of side effects are major issues with oral capecitabine treatment and provide some explanation about the patients’ behavior. Future studies should confirm those results and evaluate the role of tailored therapeutic educational interventions. Patients are looking forward for such interventions, driven by their healthcare providers.

## Availability of supporting data

The data sets supporting the results of this article are included within the article (and its additional file).

## Additional file

**Additional file 1:** The questionnaire used. More information on methods. More results from the interviews. **Figure S1** explaining the MCA and HCA process. **Figure S2** showing the standard deviation around intakes per patient (in hours), estimated from the MEMs data. **Table S1** results of the content analysis of the interviews.
