# Supplementary material for: Adherence to oral anticancer chemotherapy: What influences patients’ over or non-adherence? Analysis of the OCTO study through quantitative–qualitative methods
Source: BMC Res Notes. 2015 Jul 4;8:291. doi: 10.1186/s13104-015-1231-8 (PMC4490730; doi:10.1186/s13104-015-1231-8)
Supplement: Supplementary file 1 — Additional file 1: The questionnaire used. More information on methods. More results from the interviews. Figure S1 explaining the MCA and HCA process. Figure S2 showing the standard deviation around intakes per patient (in hours), estimated from the MEMs data. Table S1 results of the content analysis of the interviews. [file 13104_2015_1231_MOESM1_ESM.doc]

# Additional file 1

## Questionnaire

Q1: How old are you?

Q2: Man or Women

Q3: where do you live in? Town or Country

Q4: with whom do you live? Couple alone – couple with children – alone

Q5: do you have children? Yes/no

Q6: what is your educational level? Primary school/secondary school/ College

Q7: actually you are: retired/working/on sick leave / at home / unemployed

Q8: what is your last occupation: farmer/ trader/ executive / employee/ labourer / housekeeper?

Q9: did you received other cancer treatments before Capecitabine ?

Q10: how many chemotherapies did you get before Capecitabine ?

Q11: do you regularly take other treatment related to the cancer?

Q12: do you regularly take other treatment related to side effects?

Q13: do you regularly take other treatments for others medical conditions?

Q14: did you know there were oral chemotherapies before Capecitabine was prescribed to you?

Q15: Did you know you had the choice between Intraveinous treatment and Capecitabine ?

Q16: what are the main advantages of oral chemotherapy according to you (compared with IV)?

Q17: what are the main disadvantages of oral chemotherapy according to you (compared with IV) ?

Q18: your oncologist gave you sufficient explanations about the treatment (organisation, administration) : yes/no

Q19: your oncologist gave you sufficient explanations about the treatment side effects occurrence: yes/no

Q20: your oncologist gave you sufficient explanations about the treatment side effects management: yes/no

Q21: did you ask something more to your oncologist about Capecitabine first prescription?

Q22: can you tell me, how many times, how many pills of and when do you have to take your treatment?

Q23: do you know how the dose is calculated?

Q24: Do you have the intention to gather more information elsewhere? Yes/no

Q25: if yes where?

Q26: do you intend to organise your everyday life for your treatment?

Q27: according to you, what are the side effects that can occur?

Q28: what are the side effects you fear more?

Q29: what do you intend to do if one side effect occurs?

Q30: could you miss one dose during the treatment?

Q31: could you stop voluntarily the treatment?

Q32: do you think that missing a dose is dangerous?

## method:

*Bias:*

All consecutives patients were included in the study. MEMS method of adherence estimation was assigned randomly to half of included patients. Questionnaires were self-reported, interviews were conducted by the same sociologist, and MEMS results were not communicated to patients. Interviews were proposed to all patients, without knowledge of discontinuation of capecitabine or not.

*Sample size*

Sample size was determined for the genuine OCTO study and extracted from a previous pharmacokinetic study [19] : 40 patients, among which 20 with a MEMS adherence measurement, were expected for the pharmacokinetic modeling.

## Results :

*Interviews results – declared adherence, side effects occurrence and management*

Eight of the 16 patients stopped the treatment before the 6 cycles prevision because of toxicity or tumor evolution; eight completed the planned six treatment cycles.

Among the 16 responders, 14 declared they had never missed a dose and 2 (12.5%) said it happened once.

The 16 patients (100%) declared having experienced side effects. 8 (50%) declared significant side effects with no change in dose, 2 (12.5%) declared significant side effects and had a dose reduction in consequence and 6 declared light side effects (37.5%).

All patients declared skin problems: they used lotions to lower them but none had standardized protocol given by their clinicians: they bought lotions recommended by another patient, or by the pharmacist …10 (62.5%) patients had diarrhea : one of them was hospitalized and 3 had diarrhea resistant to symptomatic treatments. 7 (43.8%) patients complain of vomiting, 4 (25%) reported Hand-Foot syndrome and used the same random lotions as above.

**Figure S1 A: MCA factor MAP**


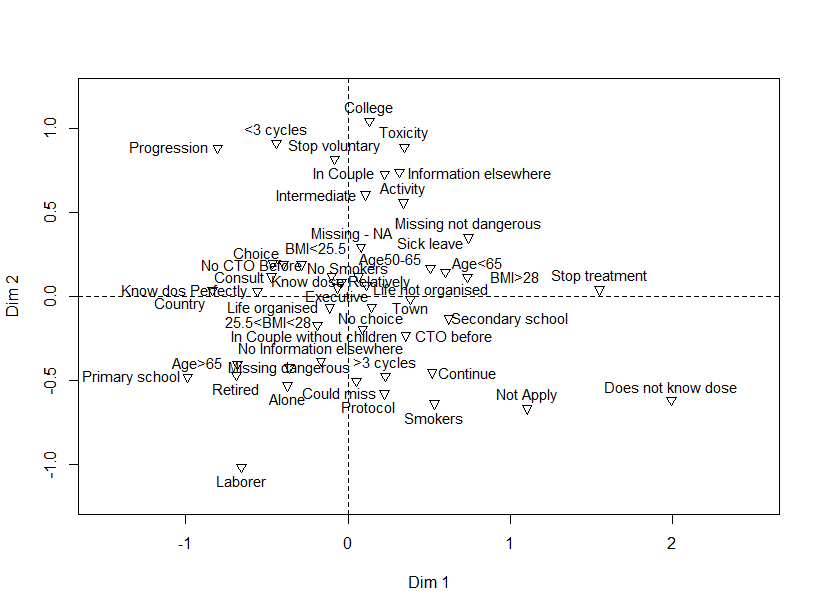


**Figure S1 B : Hierarchical clustering analysis**


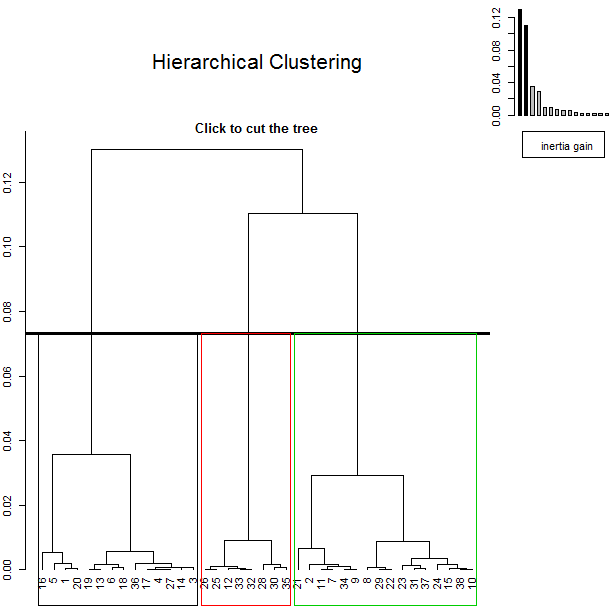


**Figure S2 :MEMS data : standard deviation around intakes per patient (in hours)**

**SD for the morning intake : 1.003 SD for the evening intake : 1.06**

**Table S1:** results of the content analysis of the interviews

| **Topics addressed**  **N=16** | **Patients profiles** |  |
| --- | --- | --- |
| **Capacitabine administration management ( 12 hours delay, meals..)** | Patients with an extremely regular way of life | Patient with an irregular way of life |
|  | It is now an habit, routine | Treatment is experience as very constraining |
|  | Adjustments are well tolerated but implies a great rigor toward the clock | Adjustments are chaotic |
|  | For both group of patients, instructions were first strictly followed and after few weeks, adjusted to the way of life | |
| **Relatives’ place in treatment adherence** | Patients with a close and available relative | Patients with distant or busy and young relatives |
|  | The relative is watchful toward the intake, plays the reminder. He/she is always around | The patient feels lonely and endorses the whole responsibility of the treatment |
|  | The sharing of responsibility reduces the anxiety | The loneliness improves the anxiety and avoids an unfazed adherence |
| **Satisfaction towards the capecitabine** | Patients satisfied with the ambulatory aspect | Patients who would rather like an hospital IV administration |
|  | They prefer to live a normal life, away from hospital | They refer to let the disease (and the treatment) at the hospital |
|  | Patients with no anxiety related to the treatment | Anxious patients who appreciate the contact with health care professionals |
|  | In general, patients have no criticism against the treatment, their opinions being to dominated by the expectation related the capecitabine efficacy | |
| **Side effects experiences** | All patients experienced side effects. What predominates is the fear of dose reduction and then efficacy reduction. So patients stand for side effects and develop numerous strategies to tolerate them. Sometimes beyond reasonable and safe. | |
